# Nicotine Gateway Effects on Adolescent Substance Use

**DOI:** 10.5811/westjem.2019.7.41661

**Published:** 2019-08-20

**Authors:** Michelle Ren, Shahrdad Lotfipour

**Affiliations:** * University of California, Irvine, Department of Pharmaceutical Sciences, Irvine, California; † University of California, Irvine, Department of Emergency Medicine and Pharmaceutical Sciences, Irvine, California

## Abstract

Given the rise in teenage use of electronic nicotine delivery systems (“vaping”) in congruence with the increasing numbers of drug-related emergencies, it is critical to expand the knowledge of the physical and behavioral risks associated with developmental nicotine exposure. A further understanding of the molecular and neurochemical underpinnings of nicotine’s gateway effects allows emergency clinicians to advise patients and families and adjust treatment accordingly, which may minimize the use of tobacco, nicotine, and future substances. Currently, the growing use of tobacco products and electronic cigarettes among teenagers represents a major public health concern. Adolescent exposure to tobacco or nicotine can lead to subsequent abuse of nicotine and other substances, which is known as the gateway hypothesis. Adolescence is a developmentally sensitive time period when risk-taking behaviors, such as sensation seeking and drug experimentation, often begin. These hallmark behaviors of adolescence are largely due to maturational changes in the brain. The developing brain is particularly vulnerable to the harmful effects of drugs of abuse, including tobacco and nicotine products, which activate nicotinic acetylcholine receptors (nAChRs). Disruption of nAChR development with early nicotine use may influence the function and pharmacology of the receptor subunits and alter the release of reward-related neurotransmitters, including acetylcholine, dopamine, GABA, serotonin, and glutamate. In this review, we emphasize that the effects of nicotine are highly dependent on timing of exposure, with a dynamic interaction of nAChRs with dopaminergic, endocannabinoid, and opioidergic systems to enhance general drug reward and reinforcement. We analyzed available literature regarding adolescent substance use and nicotine’s impact on the developing brain and behavior using the electronic databases of PubMed and Google Scholar for articles published in English between January 1968 and November 2018. We present a large collection of clinical and preclinical evidence that adolescent nicotine exposure influences long-term molecular, biochemical, and functional changes in the brain that encourage subsequent drug abuse.

## INTRODUCTION

The growing use of tobacco and electronic nicotine delivery systems (“vaping”) among teenagers represents a major public health concern. Smoking is not only the leading cause of preventable death worldwide, but epidemiological, clinical, and preclinical data have also shown that adolescent exposure to tobacco or nicotine can lead to subsequent abuse of nicotine and other substances.^1^–^19^ This phenomenon is known as the gateway hypothesis.^10^,^20^,^21^ Furthermore, adolescents are more likely to first experiment with combustible cigarettes and/or e-cigarettes than they are marijuana.^22^,^23^ Sequence patterns of drug initiation were examined in a recent study (2015), which reported that 38.8 percent of adolescents initiate nicotine before alcohol and/or marijuana, while 21.3 percent use alcohol prior to nicotine and/or marijuana, and 8.6 percent use marijuana before nicotine and/or alcohol.^23^

Although previous reports highlight that the rates of cigarette smoking are decreasing in the United States (U.S.), from 20.9 percent in 2005 to 15.5 percent in 2016, current trends in teen use of electronic nicotine delivery systems (e.g., e-cigarettes, vaporizers, hookah pens) are rapidly increasing.^24^–^26^ In particular, the rate of current e-cigarette use in high school students jumped from 1.5 percent in 2011 to 11.7 percent in 2017, then alarmingly to 20.8 percent in 2018.^24^,^27^ Among middle school students, a rise of 48 percent in e-cigarette use has also been reported from 2017 to 2018. This translates to a massive surge of an additional 1.5 million youth having been exposed to e-cigarettes in the last year alone in the U.S. The youth are often attracted to e-cigarettes due to their flavoring, easy availability, and a lack of awareness of their harmful effects.^28^,^29^ While e-cigarettes are marketed to aid in smoking cessation for adults, they have had inconsistent effects on cessation in adults and have been shown to promote smoking progression in the youth, with increased cigarette smoking in adolescents who had previously used e-cigarettes (19.1 percent) compared to those who had not (4.6 percent).^30^,^31^ In this review, we present studies that support a causal role of adolescent nicotine exposure in maladaptive alterations in reward processing during and beyond adolescence, with molecular, neurochemical, and cognitive impacts on the brain that ultimately encourage subsequent drug use.

Adolescence is a period of transition characterized by significant hormonal, psychosocial, and neural changes in rodents (postnatal day (PND) 28–42) and humans (12–18 years of age).^32^ Adolescence is a time of increased exploration and the development of social, emotional, and cognitive skills to prepare for independence of adulthood. However, adolescence is also associated with increased vulnerability to stress and risk-taking behaviors, such as sensation seeking and experimentation with recreational drugs.^33^–^35^ These age-specific behaviors are largely due to maturational changes in the brain.

During this sensitive maturational period, the brain is particularly vulnerable to the harmful effects of drugs of abuse, including tobacco and nicotine products. Nicotine is the primary psychoactive constituent in tobacco products and binds to nicotinic acetylcholine receptors (nAChRs), which are pentameric ligand-gated ion channels composed of α and β subunits (α1–7, 9–10; β1–4). nAChRs are widely distributed throughout the human and rodent brain and periphery, and are critical in the processes of the neuromuscular junction, neurotransmitter release, brain maturation, reward processing, and cognition.^36^–^45^ nAChRs are activated endogenously by acetylcholine or exogenously by nicotine, and are expressed by the majority of neuronal subtypes, including dopaminergic neurons, which facilitate drug intake and abuse.^46^–^49^ Nicotine exposure during adolescence, in particular, disrupts the normal development and expression of neuronal nAChRs, ultimately altering the function and pharmacology of the receptor subunits and changing the release of dopamine, serotonin, GABA, glutamate, and other reward-related neurotransmitters.^50^–^52^

Many factors are recognized to contribute to the onset of teenage substance abuse, such as genetics, stress, and socioeconomic status.^53^,^54^ While various mechanisms may impact substance abuse and addiction, this review focuses on the influence of developmental nicotine exposure on long-term changes in reward neural circuitry and subsequent drug use. We highlight findings from both human and rodent studies, as animal models provide insight into human brain maturation, physiology, and behavior.^32^,^55^,^56^ We argue that the effects of nicotine are highly dependent on timing of exposure, and that nAChRs interact with other drug receptor systems to directly mediate reward and reinforcement.

**Population Health Research Capsule**

What do we already know about this issue?

*Adolescent initiation of nicotine products is associated with future substance use, and teenage use of electronic nicotine devices (“vaping”) is rapidly escalating*.

What was the research question?

*We performed a thorough review of the literature to characterize impacts of nicotine on the adolescent brain*.

What was the major finding of the study?

*Nicotine triggers changes in the adolescent brain that alter reward processing and encourage future drug use*.

How does this improve population health?

*Increasing collaboration, resources, and education about the risks of teen nicotine use may contribute to decreases in addiction and drug-related emergencies*.

### Clinical Implications

The escalation in teenage use of nicotine products prompts the need to raise awareness of the detrimental effects of developmental nicotine exposure. A more complete understanding of nicotine’s gateway effects during adolescence is critical due to the extremely high and rising economic and societal costs, as well as deaths, associated with substance use. Estimates suggest that drug dependence in the U.S. is associated with over $700 billion in annual costs and more than 64,000 drug overdose deaths in 2016, which is nearly double what was observed the prior decade and continues to climb.^57^–^59^ We provide evidence for the gateway hypothesis in an effort to build knowledge for Emergency Department clinicians and other healthcare professionals to exhaustively advise their patients and patients’ caretakers. The depth of this understanding, specifically the molecular consequences of adolescent nicotine use, allows for individualized treatment plans with a greater emphasis on medication interactions, care coordination, community resources, education, and advocacy. These clinical adjustments may contribute to decreases in addiction and drug-related emergencies.

## METHODS

Prior to drafting this manuscript, the two authors independently evaluated and summarized research articles that addressed adolescent substance use and nicotine’s impact on the developing brain and behavior. We conducted a comprehensive review of the literature using a two- to three-word combination of the following keywords: adolescence, substance use, nicotinic acetylcholine receptors, gateway, reward, smoking, tobacco, nicotine, alcohol, psychostimulant, cocaine, amphetamine, cannabis, opioids. We utilized the electronic databases of PubMed and Google Scholar for research articles published in English between January 1968 and November 2018. Articles were included in the review if they discussed nicotine exposure during adolescence, drug sequence patterns, or adolescent substance use. The references from relevant articles and websites of relevant organizations were also examined for other potential sources of information. Out of 80,000 initial search results, approximately 5,000 were reviewed as relevant and non-duplicate articles. To retain focus on adolescent initiation of nicotine products, studies related to maternal tobacco or nicotine exposure were excluded. Studies evaluating other interventions (i.e., medication, sleep, exercise) were also excluded to maintain focus on nicotine’s effects on brain function and behavior. We grouped studies together according to their methodological similarities, so findings without substantial support or reproducibility (i.e., fewer than 5 comparable studies) were excluded. Following exclusion and careful analysis of studies based on key results, limitations, suitability of the methods to test the initial hypothesis, and quality and interpretation of the results obtained, 174 references were selected. The use of two reviewers and two extensive electronic databases allows for a widespread range of research articles, which maximizes scientific credibility and minimizes potential bias.

## RESULTS

### All Drugs of Abuse Share a Final Common Brain Pathway

Drugs of abuse provide rewarding, pleasurable feelings that contribute to its reinforcement (i.e. repeated use). Reward and reinforcing efficacy are measured in animals with drug self-administration on fixed and progressive ratio schedules of reinforcement, intracranial self-stimulation, oral intake, inhalation, and/or conditioned place preference. Although common drugs of abuse, like marijuana, cocaine, alcohol, and opioids, act on different neurotransmitter systems, they all exert their reinforcing effects via the mesolimbic pathway, a dopaminergic pathway that connects the ventral tegmental area to the nucleus accumbens.^60^–^66^ The development, projections, and functions of this pathway are strongly influenced by acetylcholine, glutamate, serotonin, and GABA.^67^–^71^ Dopamine release into the nucleus accumbens regulates motivation and desire for rewarding stimuli and facilitates reward prediction.^72^,^73^ As nAChRs modulate dopamine release, the gateway hypothesis posits that adolescent nicotine exposure primes the brain’s reward system to enhance the reinforcing efficacy of drugs of abuse.^74^–^77^

### Nicotine Uniquely Activates the Adolescent Brain Reward System

Substantial epidemiological data suggest that teenagers are more vulnerable than adults to nicotine dependence following minimal tobacco exposure (fewer than seven cigarettes in one month), and individuals who begin smoking during adolescence are more likely to experience difficulty quitting than those who start as adults.^78^–^84^ Indeed, 90 percent of adult smokers started before age 18.^34^,^59^ Event-related functional neuroimaging studies in children, adolescents, and adults suggest that children and adolescents have over-reactive reward responses and improved task performance when earning rewards, suggesting enhanced engagement in behaviors that result in immediate gratification.^85^ Such factors make adolescents more vulnerable to drug use and abuse.

**Figure 1:**
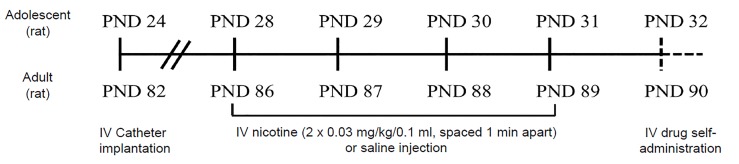
4-day nicotine pretreatment paradigm in testing the nicotine gateway hypothesis in rats. Two intravenous nicotine (0.03 mg/kg/0.1 ml, equivalent to 1–2 cigarettes) or saline injections, spaced one minute apart, are administered daily for 4 consecutive days during early adolescence (PND 28–31) or adulthood (PND 86–89). Experimentation following nicotine pretreatment (dashed lines) varies upon the drug administered, duration of drug administration, and contingent or non-contingent injections. The daily nicotine dose yields peak serum levels of approximately 30 ng/ml in both adolescents in adults, which is well within the range of the average smoker. *PND*, postnatal day*; IV*, intravenous; *mg*, milligram; *kg*, kilogram; *ng*, nanogram; *ml*, milliliter.

Animal models allow for experimenter-controlled administration of nicotine and investigation of its direct consequences on the brain and behavior through neuroimaging, biochemical assays, and behavioral tests. Early adolescent rats exposed to intravenous nicotine levels equivalent to one to two cigarettes per day for four days (Figure 1) self-administer a greater amount of cocaine, methamphetamine, and alcohol compared to adolescent rats not exposed to nicotine, as well as compared to exposed and unexposed adults.^86^,^87^ These data strongly suggest that adolescent nicotine use increases the reinforcing effects of other drugs. In addition, adolescent, but not adult, rodents exposed to nicotine display disruptions in hippocampal learning, long-lasting depressive phenotypes, changes in cocaine sensitivity and reward, enhanced drug-related learning, and deficits in impulse control, executive function, and cognition.^86^,^88^–^94^ Improved drug-related learning following brief nicotine exposure during early adolescence is characterized by rapid initiation and cue association of cocaine and amphetamine self-administration, which is indicative of an addictive-like phenotype and is not observed in adolescent and adult controls or adults also pretreated with nicotine.^92^,^94^ Furthermore, heightened depressive- and anxiety-like behaviors after 30 days of nicotine abstinence in mice exposed as adolescents, but not adults, indicate that nicotine exposure and withdrawal can have long-term effects on emotional and cognitive functioning, particularly when nicotine exposure occurs during adolescence.^89^ The exact timing of exposure during adolescence is also significant, as nicotine’s effects are far greater during early adolescence (PND 28–31 or 12–15 years) versus late adolescence (PND 38–41 or 16–18 years) or adulthood (PND 86–89).^86^,^95^ Behavioral alterations brought on by developmental nicotine exposure are driven by molecular mechanisms, including epigenetic influences, synaptic activity, and receptor signaling and regulation.^8^,^96^,^97^ Adolescent, but not adult, nicotine exposure in rodents results in the expression of distinct subunits of nAChRs (α5, α6, and β2) and persistent nAChR upregulation in the midbrain, cerebral cortex, and hippocampus.^98^,^99^ Due to the role of nAChRs in neurotransmitter release and reward processing, alterations in their quantity and function influence reward behavior. In addition, brief nicotine exposure in early adolescent rats enhances cellular activity, dopamine D2 receptor signaling, and serotonin 5-HT receptor function in brain reward areas compared to adult rats also exposed to nicotine.^86^,^90^,^100^ Moreover, chronic nicotine exposure during, but not after, adolescence alters gene expression in the ventral tegmental area and stimulates hyperresponsiveness of dopaminergic nerve terminals in the medial prefrontal cortex.^93^,^101^,^102^ These nicotine-induced changes in reward-related neurotransmitters and brain regions during adolescence may contribute to alterations in reward regulation and behavior.

The changes in brain function and behavior from developmental nicotine exposure are long lasting and a consequence of manipulation of the brain’s reward network, including the prefrontal cortex, nucleus accumbens, ventral tegmental area, hippocampus, and basolateral amygdala.^20^ Specifically, adult rodents exposed to nicotine as adolescents show a persistent increase in deltaFosB in the nucleus accumbens, impaired GABA signaling in the ventral tegmental area, and changes in brain morphology and gene expression in reward regions.^93^,^101^,^103^–^105^ Furthermore, adult rodents exposed to nicotine as adolescents have an increased preference for cocaine, amphetamine, opioids, and higher doses of nicotine.^103^ The following section reviews in greater detail the impacts of adolescent versus adult nicotine exposure on subsequent drug use in animal models. Other drug-associated behaviors are beyond the scope of this review and will not be discussed.

### Adolescent Nicotine Exposure Increases Alcohol Consumption

The developments of alcohol and tobacco use patterns are closely related among teenagers, but the order of progression is not universal among cultural and ethnic demographics.^106^ Alcohol and nicotine products are more frequently co-abused than consumed separately, as a survey of high school seniors revealed that 88 percent of smokers were drinkers, while 55 percent of nonsmokers were drinkers.^107^,^108^ However, tobacco use predicts subsequent alcohol use better than the reverse.^106^ Individuals who initiate smoking before age 17 are at a higher risk of alcohol abuse and dependence than those who begin after 17.^109^–^111^ These studies lead to the hypothesis that adolescent exposure to nicotine may lead to enhanced alcohol intake later in life.

Adolescent susceptibility to co-use of nicotine and alcohol is also observed in rodents, as concurrent self-administration of both drugs in adolescent, but not adult, rats is reinforcing and leads to an increase in subsequent oral alcohol intake.^112^ Moreover, a different nicotine exposure paradigm promotes long-lasting increases in alcohol self-administration exclusively in nicotine-treated adolescents.^104^ Nicotine exposure during adulthood can also change subsequent alcohol consumption, which indicates the influence of nicotine on alcohol reward and reinforcement; however, enhanced alcohol intake is more likely to occur if nicotine is administered prior to alcohol access.^113^ These findings collectively indicate that nicotine exposure during adolescence enhances alcohol consumption more than if the same exposure occurs later in life.

### Adolescent Nicotine Exposure Increases Psychostimulant Reinforcement and Reward

In humans, adolescent exposure to nicotine influences the likelihood of other psychostimulant use, including cocaine and methamphetamine.^3^,^5^,^8^ Data from a 1994 National Household Survey on Drug Abuse report that individuals who smoked cigarettes before age 15 were up to 80 times more likely to use illegal drugs than those who did not, with cocaine being the most likely drug to be used among young cigarette smokers.^5^ A separate study of a cohort representative of the U.S population revealed that the rate of cocaine dependence was highest among cocaine users who initiated cocaine after having smoked cigarettes (20.2 percent), and the rate of dependence was much lower among those who initiated cocaine before smoking (6.3 percent).^8^

Preclinical studies also demonstrate associations between adolescent nicotine exposure and psychostimulant consumption. Chronic nicotine exposure differentially alters cocaine-induced locomotor activity and intravenous cocaine self-administration in adolescent versus adult rodents.^103^,^114^–^116^ Adolescent rats exposed to nicotine become considerably more sensitized to the locomotor-activating effects of cocaine compared to non-exposed adolescents.^115^ Nicotine exposure during adolescence, but not adulthood, also encourages increased self-administration of cocaine during adulthood, suggesting that nicotine use may carry a greater risk during adolescence than adulthood.^116^ The effects of adolescent nicotine pretreatment on psychostimulant reinforcement and locomotor activity are mediated by nAChRs (α7 and α4β2) and serotonergic (5-HT1A) receptors.^86^ In addition, chronic and sub-chronic nicotine-exposed adolescent rats experience greater preference for and self-administration of cocaine and methamphetamine versus saline-exposed rats.^86^,^87^,^117^,^118^ Pre-adolescent nicotine exposure in rats also leads to increased cocaine-primed reinstatement, a model of relapse behavior.^119^ In contrast, alcohol pre-exposure in rats does not influence subsequent cocaine self-administration or cocaine relapse behavior, highlighting the unique gateway effects of nicotine on psychostimulant use.^120^

### Nicotine Interacts With the Endocannabinoid System

In addition to the enhanced use of alcohol and psychostimulants following early nicotine use, cigarette smoking in adolescents and young adults is associated with earlier onset of cannabis use, more frequent cannabis use, and a larger number of cannabis use disorder symptoms compared to those who did not smoke cigarettes.^9^,^121^,^122^ Likewise, teens who use e-cigarettes or hookah are more than three times more likely to use marijuana, and cannabis users report that nicotine enhances the pleasurable effects of tetrahydrocannabinol (THC), the main psychoactive constituent of marijuana that exerts its effects via cannabinoid receptors.^19^,^123^ The endocannabinoid system, which comprises cannabinoid receptors (CB1 and CB2) and endogenous ligands (anandamide and 2-Arachidonoylglycerol) throughout the central and peripheral nervous system, plays an important role in cognition, learning and memory, pain relief, emotion, stress, and reward processing.^124^,^125^

Although little research has been done on nAChRs interactions with THC specifically during adolescence, preclinical findings in adults suggest that cholinergic and endocannabinoid systems interact to modulate reward-related processes.^126^–^128^ Selective antagonism of α7 nAChRs in rats blocks the discriminative effects of THC and reduces intravenous self-administration of a cannabinoid CB1 receptor agonist (WIN55,212-2).^129^ This association appears to be bidirectional, as blockade of CB1 receptors reduces nicotine self-administration in rats.^130^,^131^

THC impacts adolescents and adults distinctively, where adolescent rats experience less of THC’s anxiogenic, aversive, and locomotor-reducing effects than adult rats.^132^ Nicotine also facilitates THC’s hypothermic, antinociceptive, and hypolocomotive effects in mice.^126^ Sub-chronic nicotine exposure in adolescent rats induces long-lasting effects in cannabinoid CB1 receptors, including increases in the hippocampus and decreases in the striatum.^133^ The association between nicotine and cannabis use and the role of reward processing in both the cholinergic and endocannabinoid systems encourages the hypothesis that nicotine may encourage and perpetuate cannabis use.

### Nicotine Interacts With the Opioidergic System

The endogenous opioid system is primarily involved in pain relief, reward processing, emotion, stress, and autonomic control, and consists of 3 families of receptors: mu, delta, and kappa.^134^ Opioid receptors located in the brain and periphery are activated endogenously by enkephalins, dynorphins, endorphins, and endomorphins, as well as exogenously by opioids (e.g., heroin, morphine, oxycodone, fentanyl). Enkephalins, endorphins, endomorphins, and opioids act primarily through mu opioid receptors (MORs) to reduce pain perception, while dynorphins preferentially act at kappa opioid receptors (KORs) to regulate appetite, stress, and emotion. Mu and delta opioid receptors play a critical role in drug reward, whereas the KORs participate in drug aversion.^135^–^137^

Although opioid use has not been extensively evaluated during adolescence, an abundance of clinical and preclinical evidence suggests an important bidirectional relationship between nicotine use and opioid reward.^136^ There is a significant overlap in the distribution of neuronal nAChRs and opioid receptors. Activation of nAChRs can influence excitability of opioid-containing neurons, and nicotine-induced dopamine release in the nucleus accumbens is dependent on activation of MORs in the ventral tegmental area.^138^–^140^ Furthermore, nicotine induces a release of endogenous opioids in the brain, and repeated exposure to nicotine can alter expression and/or functioning of opioid receptors.^141^–^144^

Perhaps unsurprisingly, given the significant overlap of cholinergic and opioidergic systems, clinical data show that treatment with naloxone and naltrexone, both opioid receptor antagonists, reduces tobacco smoking and craving for tobacco smoke.^145^,^146^ In addition, opioid-dependent smokers present with more severe nicotine dependence, respond poorly to smoking cessation medications, and may have a higher risk of relapse compared to non-opioid dependent smokers.^147^–^150^

The relationship between nicotine and the opioidergic system is similarly substantial in preclinical studies, which is important given the roles of both systems in reward processing. Early adolescent nicotine exposure in mice enhances subsequent morphine reward.^151^ In addition, blocking nicotinic receptors reduces rewarding effects of morphine, and activation of MORs decreases nicotine withdrawal symptoms.^152^–^155^ MOR antagonists increase somatic withdrawal symptoms and aversion in nicotine-dependent mice and rats, and decrease nicotine self-administration, nicotine preference, and cue-induced reinstatement of nicotine seeking.^154^,^156^–^160^ However, a small number of conflicting studies report no significant differences in nicotine reward, self-administration, or withdrawal following administration of a MOR antagonist, possibly as a result of differences in route of administration, dose, duration, or pharmacodynamics of the antagonist used.^160^–^162^ Moreover, morphine exhibits significant functional interactions with nAChRs.^163^ Chronic nicotine treatment in mice enhances the effect of morphine on striatal dopaminergic pathways, thereby influencing locomotor activity and reinforcement.^164^

Although there are minimal data on nicotine and opioid interactions during adolescence, increasing evidence supports a role of the KOR system in modulating nicotine-associated behaviors. Rodent studies suggest that teen susceptibility to nicotine use is likely due to adolescents finding nicotine more rewarding and less aversive than adults.^52^,^165^–^171^ These differences in sensitivity to nicotine reward and aversion may be due, in part, to the KOR system, as activation of KORs increases aversive effects and withdrawal signs of nicotine in adult rodents, but not adolescents.^172^–^174^ Furthermore, KOR antagonists increase concurrent nicotine and alcohol self-administration in adult, but not adolescent, male rats.^112^ Given the interactions between the cholinergic and opioidergic systems in reward regulation and the alarming increases in opioid-related deaths, it is important to recognize and understand risk factors of opioid addiction, including adolescent nicotine exposure.

## CONCLUSION

We present epidemiological and clinical findings supporting the gateway hypothesis (Table 1), and emphasize that early adolescent nicotine exposure in various rodent models increases the acquisition and intake of nicotine, alcohol, cocaine, and methamphetamine; co-use of nicotine and alcohol; and the rewarding effects of nicotine, cocaine, methamphetamine, and opioids (Table 2). Although thousands of constituents make up combustible cigarettes, the animal studies highlighted in this review investigate the effects of isolated nicotine, which is more translationally relevant to electronic cigarette use than tobacco/cigarette smoking. This review emphasizes the emerging theme that nicotine hijacks the brain’s reward pathway, particularly during adolescence when the brain is rapidly maturing, by inducing long-term changes in brain chemistry and function.

Nicotine interacts with other neurotransmitter systems and as a result increases the rewarding effects of other drugs by enhanced activation of reward circuitry. Developing brains are incredibly susceptible to long-lasting changes from perturbations during maturation, leading to behavioral changes that continue into adulthood. The prevalence of nicotine use among adolescents and the extensive interactions between nicotinic receptors and drugs of abuse highlight the critical need to better understand how nicotine modulates long-term consequences on brain and behavior related to addiction vulnerability.

This comprehensive review was performed to provide insight into how teenage experimentation with nicotine can induce drastic, ongoing consequences on reward and reinforcement of other drugs of abuse. Alterations in nicotinic acetylcholine receptors are only part of what influence adolescent substance abuse, and the reasons why adolescents decide to use tobacco products and/or nicotine delivery devices need to be further studied. Recognizing adolescent nicotine use as a possible predisposition to addiction to nicotine itself or other substances may decrease illicit drug experimentation and the incidence of drug addiction. Thus, healthcare professionals should take caution when dealing with adolescents with a history of e-cigarette use and continue to inform about its risks. Given the biochemical adaptations as a consequence of adolescent nicotine exposure, physicians may take an individualized approach to treatment and provide additional resources for patients and their families. This increased education and advocacy may improve care coordination and lead to greater adherence to a discharge plan and improved clinical outcomes. Regulatory agencies should continue to establish age limits on the purchase of nicotine products, and increase education and awareness of the risks of smoking and/or vaping during adolescence.

## Figures and Tables

**Table 1:** Summary of epidemiological and clinical findings supporting the gateway hypothesis. Surveys of adolescents and/or young adults were conducted to assess gateway effects of nicotine on subsequent drug use. Details of these selected epidemiological and clinical surveys and findings are highlighted, including age, data source, data analysis, and main observation(s).

Age	Data source and analysis	Main observation(s)	Reference(s)
24–25 years (follow-up of former adolescents aged 15–16 years)	Longitudinal cohort of former New York State high school students, followed from grades 10 and 11 (ages 15.7–34.2). Detailed monthly drug use histories were obtained. The following sequence of progression was tested: alcohol, cigarettes, marijuana, other illicit drugs, and prescribed psychoactive drugs. In addition, months of use and non-use of cigarettes and cocaine were identified.	**Sequence pattern:** Cigarettes preceded marijuana use with or without initial alcohol use among women. However, in men, alcohol consistently preceded marijuana use even in the absence of initial cigarette use. Cigarettes preceded other illicit drugs among women, but not among men. **Cigarette and cocaine use:** Most cocaine users smoked cigarettes before they started using cocaine. In addition, most cocaine users started using cocaine while they were actively smoking cigarettes (i.e., within the same month).	3,5
11–16 years	Subjects were sampled from eight public schools in Milwaukee, Wisconsin. The subjects were interviewed twice, first during 1979–80 and again during 1981–82. Eighty-nine percent of those interviewed initially were re-interviewed two years later.	Cigarette use fell on a cumulative (Guttman) scale of use with other drugs (e.g., marijuana, beer, liquor, stimulants, depressants). Having tried substances lower on the Guttman scale made one significantly more likely to be using substances higher on the scale two years later. Use of cigarettes during middle or early high school significantly increased the likelihood that the subject would be using other drugs (e.g., beer, marijuana) two years later.	4
Years 12–15, 16–17, 18–25, 26–34, 35–49, 50 or over	1994 National Household Survey on Drug Abuse. Data were analyzed to clarify whether cigarette smoking has any effect on the initiation of illegal drug use.	Individuals who had smoked cigarettes were far more likely to use marijuana, cocaine, heroin, and/or crack. Those who smoked cigarettes before age 15 were up to 80 times more likely to use illegal drugs than those who did not. Cocaine was the drug most likely to be used among young cigarette smokers.	5
16–34 years	National Epidemiological Study of Alcohol Related Consequences, a cohort representative of the U.S. population. The rates of lifetime cocaine dependence were compared among three groups: 1) those who had started to use cocaine after they had started to smoke and before they had stopped smoking, 2) those who had started cocaine use before beginning to smoke; and 3) those who had ever smoked 0–100 cigarettes.	The rate of cocaine dependence was the highest among cocaine users who initiated cocaine after having smoked cigarettes. The rates of dependence were much lower among those who initiated cocaine before smoking or who had ever smoked 0–100 cigarettes.	8
11–20 years	National Longitudinal Study of Adolescent to Adult health data spanning a 14-year period. The relationship between gateway drugs during 11–20 years of age and drug use in adulthood was analyzed using generalized estimating equation regression models.	Exposure to marijuana and illegal substances during young adulthood was positively associated with illegal substance and cocaine use. Interactions between the gateway drugs and reporting high depressive symptoms in adolescence or adulthood were associated with increased use of marijuana, illegal drugs, and cocaine in early or young adulthood.	14
14–30 years	Systematic review and meta-analysis of longitudinal studies that assessed initial use of e-cigarettes and subsequent cigarette smoking. Study selection: longitudinal studies reporting odds ratios for cigarette smoking initiation associated with ever use of e-cigarettes or past 30-day cigarette smoking associated with past 30-day e-cigarette use.	E-cigarette use was associated with greater risk for subsequent initiation of cigarette smoking and past 30-day cigarette smoking.	17
14–16 years	Subjects were sampled from 10 public schools in Los Angeles, California. Students completed surveys at baseline (grade 9) and at a 24-month follow-up (grade 11). Associations of baseline e-cigarette, hookah, or combustible cigarette use with ever marijuana use (initiation), current marijuana use (past 30 days), and current dual use of marijuana and tobacco products were examined at the 24-month follow-up.	High schoolers who used e-cigarettes or hookah at baseline compared with those who did not were more likely to report initiation and current use of marijuana as well as dual use of tobacco and marijuana. E-cigarette and hookah use at age 14 years was associated with a 3.6- to 4-fold increase in the odds of initiating and currently using marijuana two years later. The use of e-cigarettes, hookah, and combustible cigarettes in early adolescence more than doubled the odds of currently using both tobacco and marijuana by mid-adolescence.	19

**Table 2:** Summary of preclinical studies supporting the gateway hypothesis. Rodent studies highlight nicotine pretreatment paradigms and subsequent observations, including nicotine treatment doses, duration of treatment, species used, age of exposure, behavior tests, and main observation(s).

Nicotine dose, route of administration, and duration	Species and age of nicotine exposure	Behavior test(s)	Main observation(s)	Reference
60μg/kg, IV, 4 days	Sprague Dawley rats, PND 28–32 vs. PND 86–90	IV self-administration of cocaine (0.5 mg/kg/inj), methamphetamine (0.02mg/kg/inj), or ethanol (1mg/kg/inj), 1 day each	Adolescent rats pretreated with nicotine had increased initial acquisition of cocaine, methamphetamine, and ethanol compared to saline-treated adolescents and both saline- and nicotine-treated adults.	86
0.03 mg/kg/0.1 ml, IV, 2/daily for 4 days	Sprague Dawley rats, PND 28–32 vs. PND 86–90	IV self-administration of cocaine (200 or 500 μg/kg/inj), 5 days	Adolescent rats pretreated with nicotine had greater reinforced responding for cocaine compared to saline controls and adults.	87
0.4 mg/kg/day, IP, 10 days	Sprague Dawley rats, PND 34–43 vs. PND 60–69	IV self-administration of nicotine (0.04 mg/kg/inj), 15 days	Animals exposed to nicotine during periadolescence self-administered more nicotine than vehicle-exposed animals and animals exposed during postadolescence.	99
0.1, 0.5, or 1 mg/kg, SC, 2/daily for either 1 (acute) or 7 (repeated) days	ICR (CD-1) mice, PND 28–34 vs. PND 50–56	CPP for cocaine (1, 5, or 10 mg/kg, i.p.), morphine (5 mg/kg, s.c.), and amphetamine (0.2 mg/kg, s.c.,), 3 days conditioning	Adults exposed to nicotine during early but not late adolescence had increased CPP for cocaine, morphine, and amphetamine.	103
0.5 mg/kg, SC, 2/daily, 7 days	ICR mice, PND 24–30	Locomotor activity	Adults exposed to nicotine during early adolescence had enhanced cocaine-induced locomotor sensitization compared to saline-treated animals.	103
0.4 mg/kg, IP, 14 days	Long-Evans rats, PND 28–42	Operant ethanol self-administration: 8-day ethanol fading procedure (2–8% v/v)	Adults exposed to nicotine during adolescence had increased ethanol self-administration and altered GABA transmission and chloride homeostasis in the ventral tegmental area compared to adolescent and adult saline exposure and adult nicotine exposure.	104
0.1, 0.2, 0.4, 0.8 mg/kg, SC, 10 days	Wistar rats, 150 grams (age not specified)	Operant ethanol self-administration (12% v/v)	Nicotine pretreatment at a higher dose initially suppressed alcohol consumption but stimulated alcohol consumption on repeated treatment.	113
0.4 mg/kg, IP, 7 days	Sprague-Dawley rats, ~PND 30–37 vs. ~PND 60–67 (based on body weight)	Locomotor activity	Nicotine increased locomotor activity in all animals. Adolescent rats pretreated with nicotine had sensitization to nicotine-induced repetitive motion over the 7-day nicotine treatment period. Adolescent, but not adult, rats had increased amounts of cocaine-induced repetitive motion after nicotine pretreatment.	114
0.4 mg/kg, IP, 7 days	Sprague Dawley rats, ~PND 30–37 vs. ~PND 60–67 (based on body weight)	Locomotor activity, IV self-administration of cocaine (descending doses of 1.0, 0.5, 0.25, 0.125, 0.06 mg/kg/inj)	Adult rats exposed to nicotine during early adolescence were sensitized to the locomotor-activating effects of cocaine and self-administered a greater number of cocaine infusions than adolescent rats pretreated with vehicle.	116
0.4 mg/kg, IP, 10 days	Sprague Dawley rats, PND 35–44	CPP for cocaine (1 or 3 mg/kg, IP), 12 days alternating cocaine and vehicle	Adult rats that received nicotine treatment during adolescence had enhanced preference for cocaine.	117
0.16 or 0.64 mg/kg, SC, 16 days	Sprague Dawley rats, PND 35–50	IV self-administration of methamphetamine (0.05 mg/kg/inj); methamphetamine-primed reinstatement (1 mg/kg, IP)	Nicotine-exposed versus saline-exposed rats obtained more methamphetamine infusions. The high dose of nicotine had no effect on methamphetamine intake and neither nicotine dose altered methamphetamine-primed reinstatement.	118
0.1 or 0.5 mg/kg, SC, 2/daily, 7 days	ICR mice, PND 28–34 vs. PND 50–57 vs. PND 70–77	CPP for cocaine, morphine, or amphetamine	Mice treated with nicotine during early adolescence, but not late adolescence or adulthood, showed an increase in CPP for cocaine, morphine, and amphetamine later in adulthood.	151

*PND*, postnatal day; *IP*, intraperitoneal, *IV*, intravenous, *SC*, subcutaneous; *Inj*, injection, *CPP*, conditioned place preference; *μg*, microgram; *kg*, kilogram; *ml*, milligram.
